# Supplementary material for: Quality assessment of RNA in long-term storage: The All Our Families biorepository
Source: PLoS One. 2020 Dec 1;15(12):e0242404. doi: 10.1371/journal.pone.0242404 (PMC7707483; doi:10.1371/journal.pone.0242404)
Supplement: Attachment — Submitted filename: Response to Reviewers.docx [file pone.0242404.s001.docx]

**RESPONSE TO REVIEWERS**

**Reviewers' comments:**

The authors now corrected RNA quality for puritiy and quantity, and give plausible explanations for the apparent increase in purity over time. These changes are statistically significant, but not clinically relevant. "Clinically significant" should be changed for "clinically relevant", or even better for "relevant", since this is rather a technical than a clinical aspect. Passages discussion the statistically evident seeming improvements should be shortened.

Furthermore, to address RNA qualitiy, the authors included RIN numbers for a subset of samples (please change "subsample" for "subset of samples" , line 244), demonstrating no drop in overall RNA quality. Unfortunately, the storage time of the samples tested for RIN ranges only over 24 month as compared to 94 months for the purity and quantity measures. It would be nice to see RIN results at least for a small subset of samples after medium and long term storage (eg. 50 and 90 months.

**Authors’ response:**

The Authors’ thank Reviewer 1 for the additional comments.

As per request we have altered our terminology “clinically significant” to “technically relevant”, and we have decreased the discussion concerning statistical significance. Please see lines 50, 182, 204, 228, 240, and 281.

To address the second part of the comment, we have included an additional 45 RINs with a long-term storage of 111-130 months. We are unable to complete an analysis at 50-90 months, as all samples had been stored past that time.

We modeled the short-term storage (11-25 month) RINs and the long-term storage (111-130 months) RINs separately to ensure we would not introduce heterogeneity into a combined model. The most parsimonious model including all RIN data is reported, with the same conclusion as previously reported. We hope that this addition increases the reviewers confidence in the results regarding sample integrity.
