# Supplementary material for: Quality assessment of RNA in long-term storage: The All Our Families biorepository
Source: PLoS One. 2020 Dec 1;15(12):e0242404. doi: 10.1371/journal.pone.0242404 (PMC7707483; doi:10.1371/journal.pone.0242404)
Supplement: Attachment — Submitted filename: Response to Reviewers.docx [file pone.0242404.s003.docx]

# **Response to Reviewers: Revision 1**

1. **Editor’s comment:** “Please ensure that your manuscript meets PLOS ONE's style requirements, including those for file naming.”

**Authors’ response:** Thank you for the comments. To the best of the authors’ knowledge this submitted revised manuscript adheres to the PLOS ONE's style requirements. The style of in-text citation has been altered to include square brackets and are utilizing the Vancouver style for referencing. Also, the files have been renamed as suggested.

2. **Reviewer #1 comment:** “The authors Claim (in the title and conclusion) to have studied quantity and quality of RNA isolated from blood stored in PAXgene Blood RNA Tube for 7-10 years. However, they present data on RNA quantity and puritiy (Ratio 260/280 nm).”

**Authors’ response:** Thank you for your comments. We have adjusted each of the methods (line 115), and discussion (lines 244-260) surrounding RNA concentration and RNA optical absorbency to reflect when we are specifically discussing each. Also, we have relabelled each of the figures and tables to note specifically when we are discussing RNA concentration (yield) and RNA optical absorbency (purity).

3. **Reviewer #1 comment**: “Purity of RNA is no measure of quality, which should be depicted as integrity. A more or less degraded RNA - mark of pure quality - may sow perfect purity. RNA quality should therefore be assessed e.g. by BioAnalyser or - if not available - electrophoresis. To make it even better, expression levels of a set of genes or Array Analysis will give even better evidence of good oerall and mRNA quality.”

**Authors’ response:** We understand that a 260/280 ratio is a measure of purity, however our consideration of quality was to discuss both RNA concentration and purity together. To address your comment we have included an analysis on a subset of samples for which we have RNA integrity numbers. Though this is a smaller sample size (n=164) over a shorter time span (25 months), we hope that this inclusion (lines 48-51, 73, 102-104, 107-108, 111, Table 2, 193-205, Figure 6, and 251-267), along with the previous response feature analysis of RNA concentration and purity will provide sufficient evidence for our conclusions that neither the quality nor quantity of RNA decreased over storage time.

4. **Reviewer #1 comment:** “The authors demonstrate statistically significant differences in purity of RNA isolated in 2011 and 2018, with purity rising over time, which is an overvaluation of statistics. This difference is probably statistically significant, but it is not relevant. A pure nucleic acid is generally considered to have an 260/280 ratio of around 2, which is the case at both time points.”

**Authors’ response:** We agree that the observed increase in both RNA quantity and quality, though statistically significant, are not clinically important. However, we do not feel that this is an overvaluation of statistics, rather than this one piece of statistical evidence provided by the regression model output is driven primarily by an increase in precision of the estimates, and when the data is assessed visually through figures 4-6 this increase in precision can be seen. We have mentioned both in the results section (lines 165-166, 186-187, 204-205), as well as in the discussion section (lines 217, 244-260) that while the analyses show a statistically significant positive association between storage time and both purity and concentration, this association is not clinically significant. However we can conclude that there was no decrease in purity, concentration, or RIN over time.

5. **Reviewer #1 comment:** “The conclusions the authors draw from their results are correct: RNA quantity and purity do not change with storage time of blood samples under the given conditions. But before concluding that the remaining samples show unchanged good quality for further studies this should be demonstrated by methods mentioned above.”

**Authors’ response:** At the request of the reviewer we have included further analysis on a subset of samples analysing the association between storage time and RIN (lines 48-51, 73, 102-104, 107-108, 111, Table 2, 193-205, Figure 6, and 251-267). However, we are unable at this time to assess the RIN after the same amount of storage as we were assessing with purity and yield due to logistic constraints.

6. **Reviewer #2 comment:** “The RNA concentration and purity are measured on simple nano-drop; which are not specific and usually give variable results. It is necessary to have RNA integrity number (RIN) by a bioanalyzer which. Further, correlation of RIN to RNA Quality Score (RQS) can further enhance the authenticity of the data.”

**Authors’ response:** Thank you for your comments. We have included further analysis on a subset of samples analysing the association between storage time and RIN. Though this is a smaller sample size (n=164) over a shorter time span (25 months), we hope that this inclusion (lines 48-51, 73, 102-104, 107-108, 111, Table 2, 193-205, Figure 6, and 251-267) along with the previous response feature analysis of RNA concentration and purity will provide sufficient evidence for our conclusions that the quality of RNA does not decrease over storage time. Unfortunately, the authors’ did not have access to the electropherogram trace for each of these samples, thus the height of the 18s and 28s RNA peaks, the area under the 18s and 28s RNA peaks, and the fast region area are unknown. Without these values one cannot compute the RNA quality score (Copois et al, 2007 <https://doi.org/10.1016/j.jbiotec.2006.07.032>). As the reviewer noted in the comment, RIN and RQS have been correlated, and the algorithm proposed by Schroeder et al, 2006 (doi:10.1186/1471-2199-7-3) has long been used as a gold standard of RNA quality, therefore although the authors’ recognize the value of including the RQS we feel that the inclusion of the RIN does enhance the authenticity of the data even without the RQS.

7. **Reviewer #2 comment:** ‘The conclusions drawn from the statistical analysis are biased, supporting the fact that RNA quality and purity are either better or improving with time. Although the authors themselves have mentioned that it is clinically insignificant.’

**Authors’ response:** While the authors’ agree that the systematic error leading to increased precision over time would bias the resultant estimates produced via both the response feature analysis and the linear regression, we feel that our conclusions- that the purity, yield, and RIN, do not decrease as a function of storage time- are unbiased as we clearly state in our description of the results that the resultant positive association is not clinically significant (lines 165-166, 186-187, 204-205), and in our discussion (lines 217, 244-260) we further emphasize this point noting that it is attributable to increased precision as seen through visual assessment of the models in figures 4 through 6, and potentially residual confounding. To accentuate this we have included an additional paragraph within the discussion (lines 244-260) outlining the influence of precision on the analysis and we offer our interpretation of our analysis.

# **Response to Reviewers: Revision 2**

**Reviewers' comments:**
The authors now corrected RNA quality for puritiy and quantity, and give plausible explanations for the apparent increase in purity over time. These changes are statistically significant, but not clinically relevant. "Clinically significant" should be changed for "clinically relevant", or even better for "relevant", since this is rather a technical than a clinical aspect. Passages discussion the statistically evident seeming improvements should be shortened.

Furthermore, to address RNA qualitiy, the authors included RIN numbers for a subset of samples (please change "subsample" for "subset of samples" , line 244), demonstrating no drop in overall RNA quality. Unfortunately, the storage time of the samples tested for RIN ranges only over 24 month as compared to 94 months for the purity and quantity measures. It would be nice to see RIN results at least for a small subset of samples after medium and long term storage (eg. 50 and 90 months.

**Authors’ response:**

The Authors’ thank Reviewer 1 for the additional comments.

As per request we have altered our terminology “clinically significant” to “technically relevant”, and we have decreased the discussion concerning statistical significance. Please see lines 50, 182, 204, 228, 240, and 281.

To address the second part of the comment, we have included an additional 45 RINs with a long-term storage of 111-130 months. We are unable to complete an analysis at 50-90 months, as all samples had been stored past that time.

We modeled the short-term storage (11-25 month) RINs and the long-term storage (111-130 months) RINs separately to ensure we would not introduce heterogeneity into a combined model. The most parsimonious model including all RIN data is reported, with the same conclusion as previously reported. We hope that this addition increases the reviewers confidence in the results regarding sample integrity.

# **Response to Reviewers: Revision 3**

1. **Editors comment:**There are 2 tracking-changes version in the same downlowned R2 PDF file, confusing !

**Authors’ Response**: This has been corrected.

2. **Editors comment**: Line 103, delete "a" before"another'.

**Authors’ Response**: The text has been removed.

3. **Editors comment**: Table 2: please present all values to the precision of 2 decimal points. 3 decimal points are unnecessary.

**Authors’ Response:** The table values have been changed to accommodate this request.

4. **Editors comment**: Line 50, Line 166 and Line 208, delete "technically". The word "relevant" is sufficient, and please do use the expression "technicically relevant" throughout the paper.

**Authors’ Response:** The term has been changed throughout the document.

5. **Editors comment**: Line 167, delete the confusing "(greater than for purity"

**Authors’ Response**: The text has been removed.

# **Response to Reviewers: Revision 4**

1. **Editors comment:** Page 9, line 185, the donfidence interval does not make sense : "-0.090 μg /tube (95% CI:-1.14, -0.67)", please check and correct the numbers.

**Authors’ Response**: This has been corrected to -0.90 μg /tube (95% CI:-1.14, -0.67)

2. **Editors comment:** Page 9, line 194, replace "deline" with "decline"

**Authors’ Response**: This has been corrected.

3. **Editors comment:** Page 10, line 206, replace "0.03" with "0.003"

**Authors’ Response**: This has been corrected.
